# Rubber (*Hevea brasiliensis*) seed oil toxicity effect and Linamarin compound analysis

**DOI:** 10.1186/1476-511X-11-74

**Published:** 2012-06-13

**Authors:** Jumat Salimon, Bashar Mudhaffar Abdullah, Nadia Salih

**Affiliations:** 1 School of Chemical Sciences and Food Technology Faculty of Science and Technology, Universiti Kebangsaan Malaysia, 43600, Bangi, Selangor, Malaysia

**Keywords:** Rubber seed oil, Linamarin, Toxicity, Colorimetric method, Rats, Shrimps

## Abstract

****Background**:**

The lipid fraction of rubber (*Hevea brasiliensis* (kunth. Muell)) seed was extracted and analyzed for toxicological effect. The toxicological compound such as linamarin in rubber seed oil (RSO) extracted using different solvents, such as hexane (RSO_h_), mixture of chloroform + methanol (RSO_chl+mth_) and ethanol (RSO_eth_) were also studied. Various methods analysis such as Fourier transforms infrared spectroscopy (FTIR) and colorimetric methods were carried out to determine the present of such compounds.

****Results**:**

FTIR spectrum of RSO did not show any presence of cyanide peak. The determination of cyanide by using colorimetric method was demonstrated no response of the cyanide in RSO and didn’t show any colored comparing with commercial cyanide which observed blue color. The results showed that no functional groups such as cyanide (C ≡ N) associated with linamarin were observed. Toxicological test using rats was also conducted to further confirm the absence of such compounds. RSO did not show any toxic potential to the rats. Bioassay experiments using shrimps had been used as test organisms to evaluate the toxicity of linamarin extract from RSO_h_, RSO_chl+mth_ and RSO_eth_ and LC50 were found to be (211.70 %, 139.40 %, and 117.41 %, respectively).

****Conclusions**:**

This can be attributed no hazardous linamarin were found in RSO.

## Background

Recently, production of rubber seed oil (RSO) shows a huge increase in both quantity and quality in Asia. This is because of its important role in different industrial processes. RSO is yellow in color with a semi-drying oil characteristic [1]. The oil does not contain any unusual fatty acids, and its rich source of essential fatty acids (C18:2 and C18:3) make up 52 % of its total fatty acids composition [2,3].

There is a compelling reason to explore the further commercial and pharmacological benefits of low priced and unconventional sources of RSO, such as Exploration of inexpensive sources of vegetable oils has become imperative in countries like South East Asia [4]. Chemically RSO is composed of TAG of saturated and unsaturated fatty acids. The unsaturated fatty acids are monounsaturated (oleic 18:1) and polyunsaturated such as (linoleic 18:2), or (linolenic 18:3) carboxylic acids [1].

However many studies that have been carried out in the rubber seed (RS) field indicated that the production of the RSO is facing various vital challenges and one of which is the toxin, which can lead to health problems. It is well-known that concentration of poisons may always be found in the seeds of all types of plants. One of these plants that contain toxin elements is the seeds of rubber plant [5].

**Figure 1:**
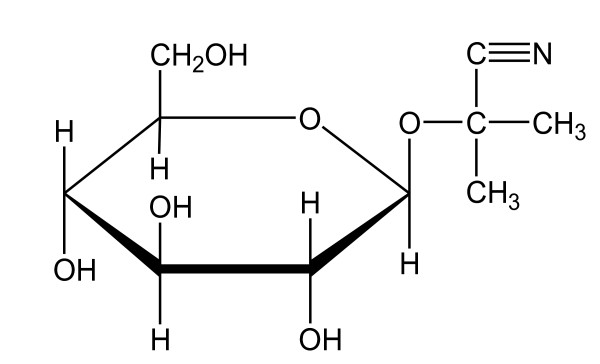
Molecular formula of linamarin.

A linamarin is a cyanogenic glucoside [6]. The molar mass of linamarin is 247.21 g mol^-1^ and the density is 1.41 g·cm^-3^. The hydrolysis or cyanogensis of linamarin by endogenous enzyme, linamarase (β-glucosidase), results in the formation of glucose and acetone cyanohydrin, which later decomposes into hydrogen cyanide (HCN) and acetone [7,8]. The molecular formula of linamarin is (C_10_H_17_NO_6_) shown in Figure 1.

The linamarin can be used as a substrate to detect the activity of enzyme linamarase it can also be used in the preparation of standard linamarin filter paper discs which are used to monitor the performance of picrate kits for determination of total cyanide, purified linamarin can also be used as an enzyme-prodrug system in cancer gene therapy [8]. The linamarin has been demonstrated to protect the plant from herbivore insect feeders [9].

In this paper, analysis the toxin of linamarin in the RSO by using different solvents extraction, such as, hexane, chloroform + methanol and ethanol were carried out. The Fourier transforms infrared spectroscopy (FTIR) analysis, colorimetric method based on the könig reaction, rats toxicological test and shrimps toxicological test were also carried out. Analysis of linamarin is playing a vital role to determine the validity of the use of the RSO as a healthy type of diet to be consumed by animal. It has various beneficial advantages to human being as well. Hence, determination of the physicochemical characteristics and toxin compound (linamarin) in the RSO are dominantly important to be scientifically researched before the RSO can be used for both animal and human consumption.

## Results and discussions

The physicochemical properties of RSO determined are given in (Table 1). The color of RSO present as pale yellow oil (33.98 ± 0.08), and darker (lower L* value) than commercial oils (66.8-68.1) such as corn oil, Soya bean oil and sunflower oil [10]. The present FFA (7.55 ± 0.02 %, as oleic acid) and acid value (15.03 ± 0.04 mg KOH/g) for RSO show that the RSO has a high FFA% due to the RSO was not neutralized. The RSO shows high iodine value (135.79 ± 0.33) comparing with iodine value of palm oil (52) [11] due to the high content of unsaturated fatty acids such as oleic acid (22.9 %) is shown in (Table 2).

**Table 1:** The physicochemical properties of RSO

**Analysis**	**RSO**
FFA% (as oleic)	7.55 ± 0.02
Acid value (mg KOH/g)	15.03 ± 0.04
Iodine value (wijs)	135.79 ± 0.33
Saponification value (mg/g)	182.12 ± 0.27
Unsaponifiable matter (%)	1.83 ± 0.01
Color	
a*	0.86 ± 0.01
b*	0.47 ± 0.04
L*	33.98 ± 0.08
Average M.wt of TAG	924.12 ± 8.89

**Table 2:** Fatty acids composition of RSO

**Fatty acids composition**	**RSO%**
Saturated	
Palmitic acid	8.56 ± 0.07
Stearic acid	10.56 ± 0.02
Total	19.12 ± 0.28
Unsaturated	
Oleic acid	22.95 ± 0.15
Linoleic acid	37.28 ± 0.10
Linolenic acid	19.22 ± 0.21
Total	79.45 ± 0.31
Others	1.43 ± 0.07

The Saponification value of RSO (182.12 ± 0.27 mg/g) similar to the other typical seed oil such as sunflower, and corn oil [12] and lower than the other vegetable oil such as, coconut, melon, groundnut, oil bean seed, and palm kernel seed, on the other hand its higher than castor [10], and *perah* oil [13], with average range saponification number of (175–250) [14]. The unsaponifiable matter is important to determine the quantity of substances present in the RSO and the quality of RSO. The value of unsaponifiable matter of RSO is 1.83 ± 0.01 % (Table 1). This value is in agreement with the value reported in by [2].

The fatty acid composition of the RSO is shown in (Table 2). The fatty acids of RSO consist from saturated FA (19.12 ± 0.28 %) and unsaturated FA (79.45 ± 0.31 %). The saturated FA are consisting mainly palmitic acid (8.56 ± 0.07 %) and stearic acid (10.56 ± 0.02 %), and unsaturated FA are consisting mainly oleic acid (22.95 ± 0.15 %), linoleic acid (37.28 ± 0.10 %), and linolenic acid (19.22 ± 0.21 %). The fatty acid composition of RSO can be used as indicator of type of each fatty acid [15].

The toxicological compound such as linamarin in RSO extracted using hexane was also carried out. The main peaks and their assignment to functional groups are given in Table 3. FTIR spectrum analysis was carried out to determine the presence of such compounds. The carbonyl band occurs as a doublet. Probably FTIR spectrum showed characteristics absorption bands of RSO at wave number (1744 cm^-1^) for the ester carbonyl functional groups (C = O). Peaks at 3009, 2924, 2925 and 2854 cm^-1^ indicated the CH_2_ and CH_3_ scissoring of RSO. The FTIR spectroscopy analysis of RSO indicated presence of sharp peaks at 1463–1413 cm^-1^ which belong to double bond (C = C) (Aliphatic). The peaks at 1284–1244 cm^-1^ of RSO referred to (C-O-C) stretching. The peaks at 1711 cm^-1^ of RSO referred to the presence of (−COOH) carboxylic acid. FTIR spectrum also showed absorption bands at 722 cm^-1^ for (C-H) group vibration. FTIR spectrum of RSO did not show any presence of cyanide peak. The result shows that no functional groups that associated with linamarin.

**Table 3:** The main peaks in the FTIR functional groups of RSO

**Wavelength of RSO**	**Functional group**
3009	O-H stretching vibration (alcohol)
2921,2854	C-H stretching vibration (aliphatic)
1744	C = O stretching vibration (ester)
1711	C = O stretching vibration (carboxylic acid)
1463, 1413	C = C bending vibration (aliphatic)
1284,1244,1166	C-O-C stretching vibration (ester)
722	C-H group vibration (aliphatic)

**Figure 2:**
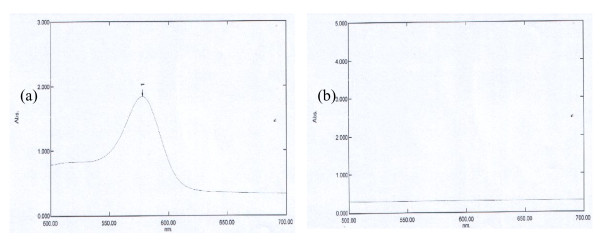
The response of cyanide (a) and RSO (b).

Two RSO were studied for cyanide determination. The RSO which was studied in this method was extracted using hexane as a solvent. The commercial cyanide was used as a standard and was compared with RSO which was extracted using hexane. The determination of cyanide demonstrated no response of the cyanide in RSO and did not show any colored comparing with commercial cyanide which observed blue color. The results of current method support the FTIR method that no cyanide observed in this measurement. The commercial cyanide showed high response at 630 nm which is reported at [16]. The colorimetric method based on könig reaction showed no response for the detection of cyanide in the RSO. The response of cyanide and RSO are shown in Figure 2.

Toxicological evaluation of the RSO was carried out in white male rats by performing an acute toxicity limit test to assess its acute toxicity potential in 3 months feeding study. Three different types of RSO were extracted by using different solvents, such as, hexane (RSO_h_), chloroform + methanol (RSO_ch+mth_) and ethanol (RSO_eth_), in the same extracting condition. Table 4 shows the mortality, color and behavior of the male white rats.

**Table 4:** Rats toxicological test: six rats

**Activity**	**Blank/Control**	**RSO_h_**	**RSO_ch+mth_**	**RSO_eth_**
Color	White	No changes	No changes	No changes
Behavior	Normal	No changes	No changes	No changes
Mortality	No	No	No	No

Note: RSO_h_: RSO was extracted using hexane as solvent, RSO_ch+mth_: RSO was extracted using mixture of chloroform and methanol as solvent, RSO_eth_: RSO was extracted using ethanol as solvent, Blank: rats food.

The results of the current study were in agreement with the above FTIR analysis. No acute toxic potential was observed with RSO. The male white rats did not display any behavioral changes and there was no mortality in any of the groups during the 3 months feeding study. The color of the male white rats didn’t show any change during this study period.

**Figure 3:**
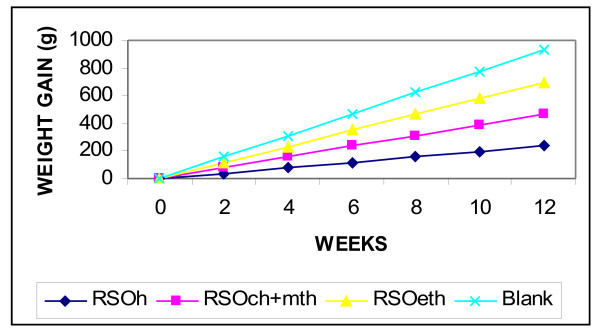
**Body weight gain of rats feed RSO and blank control. **Note: RSO_h_: RSO was extracted using hexane as solvent, RSO_ch+mth_: RSO was extracted using mixture of chloroform and methanol as solvent, RSO_eth_: RSO was extracted using ethanol as solvent, Blank: rats food.

The RSO_h_, RSO_ch+mth_ and RSO_eth_ had no adverse effect on the rats food consumption. A similar increase in the average daily gain of rats fed with RSO_h_, RSO_ch+mth_ and RSO_eth_ was also observed; however, the differences between the 3 white male rats were not statistically significant. The 3 male rats showed no significant difference in body weight gain, food efficiency, body waist measurement and body height measurement. The growth rate of 3 white male rats is shown in Table 5 and Figure 3. These results indicated that RSO_h_, RSO_ch+mth_ and RSO_eth_ had no toxic or antipalatability effects depending on the statistical analysis of p-value (*P* < 0.05) [2,17].

**Table 5:** Effect of RSO intake: six rats

**Activity**	**Blank/Control**	**RSO_h_**	**RSO_ch+mth_**	**RSO_eth_**
Initial body weight (g)	248.8 ± 0.2	248.2 ± 0.6	248.5 ± 0.7	248.1 ± 0.2
Final body weight (g)	480.1 ± 0.7	482.1 ± 0.2	480.8 ± 0.5	479.7 ± 0.3
Body weight gain (g)	232.1 ± 0.6	234.4 ± 0.6	232.8 ± 0.2	231.5 ± 0.2
Food consumption (g)	1789.8 ± 1.7	1800.2 ± 0.7	1792.6 ± 1.2	1783.9 ± 1.8
Food efficiency ratio (weight gain/ food intake)	0.15 ± 0.02	0.13 ± 0.07	0.13 ± 0.01	0.12 ± 0.03
Initial body waist (cm)	14.5 ± 0.6	14.5 ± 0.2	14.5 ± 0.1	14.5 ± 0.7
Final body waist (cm)	20.2 ± 0.2	20.5 ± 0.7	20.5 ± 0.3	20.3 ± 0.2
Body wasit gain (cm)	5.7 ± 0.6	6.0 ± 0.7	6.0 ± 0.1	5.8 ± 0,5
Initial body tall (cm)	23.4 ± 0.3	23.1 ± 0.5	23.7 ± 0.3	23.5 ± 0.7
Final body tall (cm)	24.3 ± 0.9	24.5 ± 0.2	24.2 ± 0.7	24.2 ± 0.1
Body tall gain (cm)	1.3 ± 0.7	1.5 ± 0.7	1.2 ± 0.2	1.2 ± 0.3
Condition*	Normal	Normal	Normal	Normal

Note: RSO_h_: RSO was extracted using hexane as solvent, RSO_ch+mth_: RSO was extracted using mixture of chloroform and methanol as solvent, RSO_eth_: RSO was extracted using ethanol as solvent, Blank: rats food, condition*: condition was assessed by visual appearance.

Table 6 shows the relationship between the samples of the linamarin which was extracted from the RSO using different solvents, such as RSO_h_, RSO_ch+mth_ and RSO_eth_ in the same extracting condition, concentration and the mortality rate of the shrimps. Sodium cyanide was used as a blank control in the palm oil and rubber seed (RS) tests for purposes of comparing the RSO. The estimated LC50 values and their confidence limits that resulted from the samples’ acute toxicity tests on freshwater shrimps using samples of the linamarin were extracted from the RSO_h_, RSO_ch+mth_, RSO_eth_, RS, palm oil and cyanide standard and are as listed in Table 7.

**Table 6:** **Mortality percentage of shrimps at various concentrations of linamarin samples extracted from RSO**_**h**_**, RSO**_**chl+mth**_**, RSO**_**eth**_**, RS, palm oil and NaCN**

**Conc. (mL)**	**Number exposed**	**Number response of RSO_h_**	**Number response of RSO_chl+mth_**	**Number response of RSO_eth_**	**Number response of RS**	**Number response of palm oil**	**Number response of cyanide**
5	10	0	0	0	0	0	9
10	10	0	0	0	2	0	9
50	10	0	0	0	5	0	10
75	10	1	1	1	7	1	-
100	10	1	2	3	8	2	-

Note: RSO_h_: RSO was extracted using hexane as solvent, RSO_ch+mth_: RSO was extracted using mixture of chloroform and methanol as solvent, RSO_eth_: RSO was extracted using ethanol as solvent, RS: RS extracted using water as solvent.

**Table 7:** **The estimated LC values and confidence limits of toxicity on shrimps using the linamarin samples extracted from RSO**_**h**_**, RSO**_**chl+mth**_**, RSO**_**eth**_**, RS, palm oil and NaCN**

**Point**	**Exposure conc. of RSO_h _(mg/L)**	**Exposure conc. of RSO_chl+mth _(mg/L)**	**Exposure conc. of RSO_eth _(mg/L)**	**Exposure conc. of RS (mg/L)**	**Exposure conc. of palm oil (mg/L)**	**Exposure conc. of cyanide (mg/L)**
LC 10.00	90.86	80.63	77.33	8.75	80.63	0.06
LC 50.00	211.70	139.40	117.41	40.59	139.40	0.63
LC 90.00	493.22	241.03	178.26	188.33	241.03	6.49

Note: RSO_h_: RSO was extracted using hexane as solvent, RSO_ch+mth_: RSO was extracted using mixture of chloroform and methanol as solvent, RSO_eth_: RSO was extracted using ethanol as solvent, RS: RS extracted using water as solvent.

Based on Finneys Probit Analysis Method (using EPA software program), the mean LC50 value of samples of the linamarin extracted from the RSO_h_, RSO_ch+mth_, RSO_eth_, RS, palm oil and cyanide standard using shrimps were found to be 211.70 %, 139.40 %, 117.41 %, 40.59 %, 139.40 % and 0.63 % respectively.

Shrimp has, for the first time, been used as a test organism for acute toxicity of linamarin in RSO and RS. The results showed that samples of the linamarin extract from the RSO_h_, RSO_ch+mth_ and RSO_eth_ had no toxicity effect to shrimps (LC50 211.70 %, 139.40 % and 117.41 %), as this can be attributed to the absence of hazardous linamarin in RSO. The results of the samples which were extracted from the RS showed toxic effect in shrimps LC50 was found to be 40.59 % as compared with samples which were extracted from RSO that may contain copper which produced the toxic effect [18]. The results of RSO and RS were compared with the palm oil and standard NaCN. The palm oil did not show any toxic effect with LC50 (139.40 %). NaCN contained high toxicity to the shrimps with LC50 (0.63 %). These results would indicate that RSO_h_, RSO_ch+mth_ and RSO_eth_ had no acute toxicity in shrimp’s organisms and supported the methods which used (FTIR, calorimetric method and rats toxicological test).

## Methods

### **Seed material and oil extraction**

Rubber seeds (RSs) were collected from (RRI) Sungai Buloh. The seeds were shelled and dried in the oven at 105 °C for 30 min. The RSs were milled using the grinder. The seeds were kept in the refrigerator. RSO was extracted from the 500 g RSs by soxhlet extractor using hexane as solvent at 60 °C for 6 hours.

### **Physicochemical characteristics**

The physicochemical properties of RSO such as color, FFA%, acid value, saponification value, iodine value and unsaponifiable matter were determined according to [15].

### **Gas chromatography (GC)**

The fatty acid composition of RSO was determined using its fatty acid methyl esters [19,20]. GC analysis was performed on shimadzu, GC equipped with flame ionization detectorand capillary column (30 m × 0.25 mm × 0.25 μm films). The detector temperature was programmed for 280°C with flow rate of 0.3 mL/min. The injector temperature was set at 250°C. Nitrogen was used as the carrier gas at a flow rate of 20 mL/min.

### **Fourier transforms infrared spectroscopy (FTIR)**

Fourier transforms infrared spectroscopy (FTIR) has been carried out according to [21]. FTIR of the products was recoded on a Prkin Elmer Spectrum GX spectrophotometer in the range 400–4000 cm-1. FTIR was used to measure functional groups of RSO. A very thin film of RSO was covered on NaCl cells (25 mmi.d × 4 mm thickness) and was used for analysis.

### **Colorimetric method**

#### ***Samples extraction***

The samples of linamarin extract were extracted from three different RSO (100 g) by using different solvents such as water (20 mL) and 0.25 M chilled H_2_SO_4_ (20 mL) in separating funnel. After shaking the mixture gently, the mixture was left a few minutes to get two phases oil phase, and water phase or acidic phase. The oil phase was removed and the water phase or acidic phase was kept in the fridge [22,23].

#### ***Hydrolysis of HCN in sample***

To conservation of cyanide, the samples were supplemented with (4 mL) 10 M NaOH. The sample was distilled without further pretreatment. HCN was recovered in the presence of (10 mL) zinc acetate buffer pH 4.5. The remaining cyanide was subsequently recovered by distillation after addition (5 mL) of MgCl_2_ and (5 mL) sulfamic acid plus (5 mL) 50 % H_2_SO_4_; the latter added to obtain pH 1–2 for converting to HCN during distillation. After 3 min, 45 mL 50 % H_2_SO_4_ was added and the solution boiled under reflux for 90 min [22,23]. The released HCN was determined using colorimetric method.

#### ***Determination of CN***

**Figure 4:**
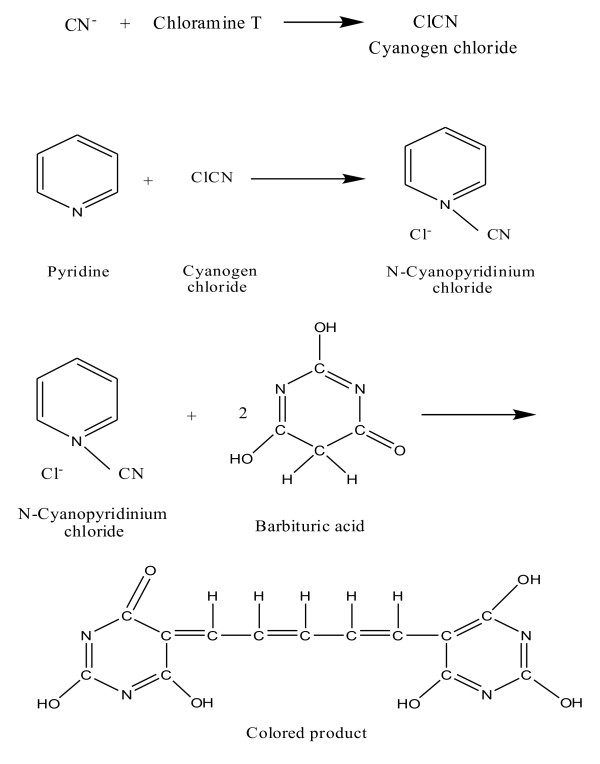
Colorimetric method reaction.

The detection of small amounts of cyanide, colorimetric method based on the könig reaction was proposed by [16]. This method involves the oxidation of cyanide to cyanogens chloride with chloramines T. Cyanogens chloride was then reacted with pyridine to form N-cyanopyridinium chloride (könig reaction). The N-cyanopyridinium chloride was then reacted with barbituric acid to produce blue color was measured between 570–630 nm [16,24]. This method was preferable for cyanide at 3.8 × 10-5 M. The reaction of the pyridine- barbituric acid method is shown in Figure 4. The same conditions were used to determine the (C ≡ N) in sodium cyanide (NaCN) which was used as a standard to compare it with HCN which was hydrolyzed from RS and RSO samples.

### **Rats toxicological test**

Male white rats (Rumah Haiwan Laboratories) weighing between 284.1-284.8 grams were used and has been carried by [17]. The male white rats were individually housed in stainless steel cages in a room with controlled temperature (30-35 °C) and lighting (alternative 12 h periods of light and darkness). The male white rats were fed a pelleted commercial laboratory for 3 months. The mortality, color, and the behavior of the male white rats were recoded daily but the food consumption was recorded every two weeks. The food consumption, food efficiency, body waist measurement and body tall measurement were also determined. Three experiments were conducted to determine the toxicological response of rats fed RSO. In experiment 1, rats were fed a RSO has been extracted by using hexane. In experiment 2, rats were fed a RSO has been extracted by using chloroform + methanol. In experiment 3, rats were fed a RSO has been extracted by using ethanol. The RSO was stored at 4 °C for feeding of rats. The toxicological evaluation of the RSO extracting by using three different solvents were carried out in male white rats by performing an acute oral toxicity limit test to assess its acute toxicity potential. The rats fed RSO were compared with rat fed normal food as blank.

### **Shrimps toxicological test**

#### ***Samples preparation***

About 100 g of the RSs were homogenized with 160 mL of water. The homogenates were filtered through a filter cloth to remove insoluble materials. The homogenizer was rinsed with the water (40 mL) which was filtered in the same way. The filtrates were put it in the dried vacuum for 2 days. The precipitation was collected and stored in the fridge (a). RSO was extracted by using different solvents (hexane, chloroform + methanol and ethanol). The samples of linamarin were extracted from three different RSO (100 g) by using water as a solvent in separating funnel. After shaking the mixture gently, the mixture was left a few minutes to get two phases oil phase, and water phase. The oil phase was removed and the water phase was kept in the fridge (b).

#### ***HCN hydrolysis***

To bind of cyanide, the samples were treated with (4 ml) 10 M NaOH. The sample was distilled without further pretreatment. HCN was recovered in the presence of (10 mL) zinc acetate buffer pH 4.5. The remaining cyanide was subsequently recovered by distillation after addition (5 mL) of MgCl_2_ and (5 mL) sulfamic acid plus (5 mL) 50 % H_2_SO_4_; the latter added to obtain pH 1–2 to release HCN during distillation. After 3 min, 45 mL 50 % H_2_SO_4_ was added and the solution boiled under reflux for 90 min [22,23]. The samples after the distillation method were put it in a drying vacuum to evaporate the solvents. The released HCN was determined using acute toxicity method.

#### ***Acute toxicity method***

Shrimps used in this study was obtained from a local breeder and transported immediately to the laboratory within 20 min. In the laboratory, a total of 840 shrimps were kept in an 80-L glass aquarium containing filtered and dechlorinated tap water (pH 6.2-6.4, dissolved oxygen concentration 7.3-8.1 mg L^-1^, conductivity 64–68 μScm^-1^ and ammonia < 0.5 mg L^-1^). The shrimp was equipped with a water-cycling device by which the water was continuously aerated for one week to remove chlorine before the shrimp was introduced. Shrimps were acclimated for 14 days (26-27 °C with 12 h light: 12 h darkness) and fed daily. Care was taken in order to keep the mortality rate less than 5 % for the whole acclimatization period. Acute toxicity test was performed according to the [25,26] recommendations. Laboratory static tests were conducted to determine the median lethal concentrations (LC50).Ten shrimps of similar size were sampled and placed in the test chambers. Shrimps were exposed for 96 hours to different samples concentrations of 5, 10, 50, 75 and 100 % of the samples which were extracted from RSO and RS. The test chambers were aerated throughout the test period. Physiochemical parameters of the water in the chambers such as pH, conductivity, dissolved oxygen, and temperature were measured for each solution. The tests were repeated three times for both the control and each test solution. During the experiment, dead shrimps were removed and mortality of the shrimps exposed to various concentrations of samples was recorded after 6, 12, 18, 24, 48, 72, and 96 hours. LC50 was calculated based on Finneys Probit Analysis Method [27]. The same conditions were used to determine the C ≡ N in sodium cyanide (NaCN) which was used as a standard to compare it with HCN, which was hydrolyzed from RS and RSO samples and was compared also with samples which were extracted from palm oil using water as solvent.

## Conclusions

The results showed that no functional groups such as cyanide (C ≡ N) that associated with linamarin being obserbed. The current study has shown that RSO could be considered for edible use. These initial results indicate that the use of RSO as an edible oil will not be restricted by toxic factors.

## Competing interests

The authors declare that they have no competing interests.

## Authors’ contributions

JS developed the concept, analyzed the data and drafted the manuscript. BMA performed the analysis of the toxin compound (linamarin) in the RSO by using different methods. NS advised on the methods of tests. All authors read and approved the final manuscript.
